# Implementation of Mental Health Service Has an Impact on Retention in HIV Care: A Nested Case-Control Study in a Japanese HIV Care Facility

**DOI:** 10.1371/journal.pone.0069603

**Published:** 2013-07-29

**Authors:** Shinjiro Tominari, Takahiro Nakakura, Toshihiko Yasuo, Kyoko Yamanaka, Yoshimitsu Takahashi, Takuma Shirasaka, Takeo Nakayama

**Affiliations:** 1 Department of Health Informatics, Kyoto University School of Public Health, Kyoto, Japan; 2 Department of Clinical Psychology, National Hospital Organization Osaka National Hospital, Osaka, Japan; 3 Department of Social Welfare, School of Humanities and Social Sciences, Osaka Prefecture University, Osaka, Japan; 4 AIDS Medical Center, National Hospital Organization Osaka National Hospital, Osaka, Japan; University of South Carolina School of Medicine, United States of America

## Abstract

**Background:**

Poor retention in the care of patients with human immunodeficiency virus (HIV) is associated with adverse patient outcomes such as antiretroviral therapy failure and death. Therefore, appropriate case management is required for better patient retention; however, which intervention in case management is important has not been fully investigated. Meanwhile, in Japan, each local government is required to organize mental health services for patients with HIV so that a case manager at an HIV care facility can utilize them, but little is known about the association between implementation of the services and loss to follow-up. Therefore, we investigated that by a nested case-control study.

**Methods:**

The target population consisted of all patients with HIV who visited Osaka National Hospital, the largest HIV care facility in western Japan, between 2000 and 2010. Loss to follow-up was defined as not returning for follow-up care more than 1 year after the last visit. Independent variables included patient demographics, characteristics of the disease and treatment, and whether the patients have received mental health services. For each case, three controls were randomly selected and matched.

**Results:**

Of the 1620 eligible patients, 88 loss to follow-up cases were identified and 264 controls were matched. Multivariate-adjusted conditional logistic regression revealed that loss to follow-up was less frequent among patients who had received mental health services implemented by their case managers (adjusted odds ratio [95% confidence interval] 0.35 [0.16-0.76]). Loss to follow-up also occurred more frequently in patients who did not receive antiretroviral therapy (adjusted odds ratio [95% confidence interval], 7.51 [3.34-16.9]), who were under 30 years old (2.74 [1.36-5.50]), or who were without jobs (3.38 [1.58-7.23]).

**Conclusion:**

Mental health service implementation by case managers has a significant impact on patient retention.

## Introduction

Patients with human immunodeficiency virus (HIV) cannot currently be free of the virus, so they must regularly visit medical facilities and receive appropriate care to prevent acquired immunodeficiency syndrome (AIDS)-related or non-AIDS-related complications. Patients who do not receive regular HIV care tend to experience adverse outcomes such as antiretroviral therapy failure, hospitalization, AIDS-defining illness, and death [1–3].

One of the approaches to preventing loss to follow-up (LTFU) and facilitating better retention is case management. It is an attempt by health care providers to connect patients to adequate ancillary services. Case management has been shown effective [4–6], but it consists of various complex activities to keep patients from LTFU and the definition of case management differs somewhat among the previous reports. Thus, it is clinically relevant to investigate which element of intervention in case management is particularly associated with these patients’ engagement in care.

Meanwhile, patients with HIV and poor mental health or psychological distress are prone to be lost to follow-up [7–9] and Japanese local governments staff therapists to provide psychotherapy at no charge as a mental health service upon requests from HIV care medical staff members. Therefore, case managers at HIV care facilities can easily utilize mental health services as resources, despite little evidence that mental health service introduction improves patient retention. Our aim is to investigate the association between mental health service implementation for patients by case managers and LTFU in HIV care.

## Methods

### Study design

We performed a nested case-control study within a cohort of patients with HIV at Osaka National Hospital, Japan.

### Setting

The population of Japan is 127 million and adult HIV prevalence is reported to be 0.017% in 2011 [10]. The Japanese national health insurance system covers HIV treatment costs. Patients with HIV have an upper monthly limit for HIV-related medical payments of 5,000 to 20,000 Japanese yen (approximately $60–240 USD at the exchange rate in 2012), which also includes the cost for treatment of opportunistic infection or malignancy. This constitutes approximately 1.5% to 6% of the average monthly salary in Japan. Some local governments provide additional monetary support. People living on welfare benefits do not have to pay any medical expenses at all.

Osaka National Hospital is a tertiary hospital and is conveniently located for transportation. It is the largest HIV care facility in the western half of Japan, providing care for half of the infected patients reported in the area as of 2010.

### Participants

The study cohort from which cases and controls were selected consisted of patients with HIV who visited the Department of Infectious Diseases at Osaka National Hospital between January 1, 2000 and September 30, 2010. Patients entered the cohort at their first hospital visit and were tracked until LTFU, death, or September 30, 2011. Patients excluded from the study were under 18 years old, did not intend to visit the hospital regularly (e.g., a tourist who needed an antiretroviral refill), or those who died within 1 year of their first visit.

### Case management with and without implementation of mental health services

All patients were attended by trained nurses as their case managers. Case managers held a 1-h intake session with each patient at his initial visit. During the session, case managers inform patients of knowledge on HIV infection and emphasized the importance of seeing a physician regularly. They assessed each patient’s unmet social, economic, or psychological needs that hindered regular visits and coordinated a care plan. According to that plan, ancillary services for the patient were implemented. For example, if the patient had monetary or housing problems, the case manager introduced a social worker. If the patient had a psychological problem that hampered the maintenance of health (e.g., anxiety, feeling of stigma, interpersonal problems, or low self-efficacy), a case manager implemented mental health services. With regard to the mental health services, the hospital was staffed with trained psychotherapists during the study period and they provided psychotherapy with a client-centered approach to the patients for free. Each therapy session was on a one-on-one basis at intervals ranging from weekly to monthly, depending on the patient’s need. Other related medical staff members were able to view records of psychotherapy on charts and communicate with the therapist. Patients were permitted to see the therapists even if they did not regularly meet with their primary physician.

### Study size

Approximately 60 cases were expected from the cohort in a preliminary investigation. The proportion of cases with independent variables was hypothesized as 20% and the proportion of controls with independent variables as 40%. With an alpha error of 5%, statistical power was calculated as 59% for 1:1 matching, 73% for 1:2 matching, 78% for 1:3 matching, and 83% for 1:4 matching. The study adopted 1:3 matching.

### Loss to follow-up cases and controls

Loss to follow-up was defined as not returning for follow-up appointments more than 1 year after the last physician visit to the Department of Infectious Disease at Osaka National Hospital, excluding emergency care and subspecialty visits (e.g., dermatology or dentistry). If a physician referred the patient to another HIV care facility for subsequent medical care at the last visit, we did not consider that case to be LTFU. All patients with LTFU were selected as LTFU cases. For each LTFU case, we identified a risk set of patients from the study cohort who remained in regular care at the time LTFU occurred. Three controls were randomly selected from each risk set whose case matched both the calendar date within 90 days of entry into the cohort and the time spent in the cohort between LTFU cases and controls.

### Variables

Independent variables were measured as either baseline variables or time-dependent variables. Baseline variables were recorded on charts by a nurse at the time of cohort entry using a standardized form. They were sex, route of infection (homosexual, heterosexual, or blood products), HIV testing motive (voluntary or provider-initiated), mental illness, illicit drug use, regular employment, welfare benefits, nationality, and key person. A key person was defined as the patient’s partner or anyone in a close relationship to whom the patient disclosed his serostatus. Time-dependent variables were evaluated as a status at index time, which was the date of last visit for LTFU cases or the corresponding date for controls. Time-dependent variables included age (<30 years or ≥30 years), CD4+ lymphocyte count (<200/mm^3^, 200-499/mm^3^, or ≥500/mm^3^), history of AIDS, antiretroviral therapy, and whether mental health service had been implemented before the index time. Acquired immunodeficiency syndrome was diagnosed according to the definition of the Japanese Ministry of Health, Labour and Welfare. All data were recorded in regular medical practice and extracted by chart review.

### Statistical analysis

All analyses conserved the matched nature of the LTFU cases and controls. Odds ratios (ORs) between each independent variable and dependent variable (LTFU) were calculated using the Mantel-Haenszel method for binominal variables and conditional logistic regression for ordinal variables. After backward stepwise variable selection with a significance level of *p*>0.20 for removal, we performed multivariate-adjusted conditional logistic regression analysis to calculate adjusted ORs. A two-sided *p*-value less than 0.05 was considered to be significant. All statistical analyses were performed using Stata 12.1 software (StataCorp LP, College Station, Texas, USA).

### Ethics statement

Personal information other than the above-mentioned variables was not included in the dataset to protect patient confidentiality. According to the Ethical Guidelines for Epidemiological Research set by the Ministry of Education, Culture, Sports, Science, and Technology with the Ministry of Health, Labour and Welfare, Japan, individual informed consent was waived because only recorded clinical data was used and additional patient data were not collected. The Kyoto University Graduate School and Faculty of Medicine Ethics Committee and Institutional Review Boards at Osaka National Hospital waived the need for written informed consent from the participants and approved the research protocol.

## Results

Of the 1715 patients with HIV in the hospital cohort, 1620 were eligible for the present study. Their mean age was 37.1 years and 1545 (95.4%) were male. The route of HIV infection was homosexual transmission in 1238 (76.4%) of patients, heterosexual transmission in 277 (17.1%), blood products in 34 (2.1%), and unidentified in 71 (4.4%).

A total of 88 LTFU cases were identified and 264 controls were selected from the cohort. The incidence of LTFU was calculated to be 1.32 per 100 person-years. The median time to LTFU was 14 months (interquartile range, 1.6-33 months). Forty LFTU cases (45.5%) resulted in absence within 1 year of the first visit. Six patients had only one hospital visit. The patients’ demographics, disease status, and treatment characteristics are summarized in Table 1. Patients in the LTFU cases had an average age of 32.7 years (standard deviation, 9.3 years) and included 86 males (97.7%). Patients in the LTFU cases were younger than the controls and had a larger proportion of patients who underwent voluntary HIV testing or who lacked regular employment. Patients in the LTFU cases also had higher CD4+ lymphocyte counts and were less likely to have an AIDS diagnosis or to have received antiretroviral therapy than the controls. Sixty-eight (91.9%) of the 74 LTFU case patients without antiretroviral therapy had CD4+ lymphocyte counts higher than the threshold for the therapy recommended by the guidelines at the time LTFU occurred or were within 90 days from their first visit. Fourteen LTFU case patients had been on anti-retroviral therapy for the median of 31 months (range, 2-80 months).

**Table 1:** Characteristics of loss to follow-up cases and controls in HIV care.

		**Loss to follow-up cases (n=88)**	**Controls (n=264)**
Age (years), mean [SD*]		32.7 [9.3]	38.5 [9.4]
**Sex, n (%)**	Male	86 (97.7%)	251 (95.1%)
	Female	2 (2.3%)	13 (4.9%)
**Mental health service, n (%)**	No	72 (81.8%)	179 (67.8%)
	Implemented	16 (18.2%)	85 (32.2%)
**Key person, n (%)**	No	20 (22.7%)	50 (18.9%)
	Yes	68 (77.3%)	214 (81.1%)
**Route of infection, n (%)**	Homosexual	72 (81.8%)	213 (80.7%)
	Heterosexual	15 (17.0%)	48 (18.2%)
	Blood products	1 (1.1%)	3 (1.1%)
**Regular employment, n (%)**	No	29 (33.0%)	46 (17.4%)
	Yes	59 (67.0%)	218 (82.6%)
**Welfare benefits, n (%)**	No	87 (98.9%)	257 (97.3%)
	Yes	1 (1.1%)	7 (2.7%)
**HIV test motive, n (%)**	Voluntary	57 (64.8%)	105 (39.8%)
	Provider-initiated	20 (22.7%)	110 (41.7%)
	Others	11 (12.5%)	49 (18.6%)
**Mental illness, n (%)**	No	84 (95.4%)	251 (95.1%)
	Yes	4 (4.6%)	13 (4.9%)
**Illicit drug use, n (%)**	No	66 (75.0%)	207 (78.4%)
	Yes	22 (25.0%)	57 (21.6%)
**Nationality, n (%)**	Japanese	83 (94.3%)	249 (94.3%)
	Others	5 (5.7%)	15 (5.7%)
**AIDS diagnosis, n (%)**	No	82 (93.2%)	195 (73.9%)
	Yes	6 (6.8%)	69 (26.1%)
**Antiretroviral therapy, n (%)**	No	74 (84.1%)	125 (47.4%)
	Yes	14 (15.9%)	139 (52.6%)
**CD4+ lymphocyte count (/mm^3^), mean [SD]**		465 [176]	367 [203]

* Standard deviation

Independent variables for LTFU are displayed in Table 2. Stepwise multivariate-adjusted conditional logistic regression revealed that the risk of LTFU in patients who had received mental health services implemented by a case manager was significantly low (0.35 [0.16-0.76], *p* = 0.008). Loss to follow-up occurred more frequently in patients who did not receive antiretroviral therapy (adjusted OR [95% confidence interval], 7.51 [3.34-16.9], *p* < 0.001), who were younger than 30 years old (2.74 [1.36-5.50], *p* = 0.005), and who lacked regular employment (3.38 [1.58-7.23], *p* = 0.002).

**Table 2:** Independent variables for loss to follow-up in HIV care.

		Crude ORs^ab^	95% CI^c^	*p*-value	Adjusted ORs^d^		95% CI		*p*-value
**Age**	≥30 years	(reference)			(reference)				
	<30 years	3.43	1.96-5.99	< 0.001	2.74		1.36-5.50		0.005
**Sex**	Male	(reference)			(reference)				
	Female	0.46	0.10-2.05	0.297	0.30		0.05-1.69		0.171
**Mental health service**	No	(reference)			(reference)				
	Implemented	0.45	0.24-0.84	0.009	0.35		0.16-0.76		0.008
**Key person**	No	(reference)			(reference)				
	Yes	0.80	0.45-1.41	0.438	0.58		0.28-1.19		0.135
**Route of infection**	Homosexual	(reference)					
	Others	0.75	0.42-1.34	0.327			
**Regular employment**	Yes	(reference)			(reference)				
	No	2.46	1.38-4.39	0.002	3.38		1.58-7.23		0.002
**Welfare benefits**	No	(reference)					
	Yes	0.43	0.05-3.48	0.414			
**HIV test motive**	Voluntary	(reference)			(reference)				
	Others	0.40	0.25-0.63	<0.001	1.50		0.38-2.70		0.180
**Mental illness**	No	(reference)					
	Yes	0.92	0.31-2.77	0.886			
**Illicit drug use**	No	(reference)					
	Yes	1.25	0.67-2.33	0.492			
**Nationality**	Japanese	(reference)					
	Others	1.00	0.32-3.09	1.000			
**AIDS diagnosis**	No	(reference)					
	Yes	0.19	0.07-0.51	0.002			
**Antiretroviral therapy**	Yes	(reference)			(reference)				
	No	8.47	3.91-18.33	<0.001	7.51		3.34-16.9		< 0.001
**CD4+ lymphocyte count**	<200/mm^3^	(reference)					
	200-499/mm^3^	3.94	1.35-11.5	0.012			
	≥500/mm^3^	6.11	2.01-18.6	0.001			

^a^ Odds ratio

^b^ Mantel-Haenszel method for binominal variables and conditional logistic regression for ordinal variables

^c^ Confidence interval

^d^ Stepwise, multivariate-adjusted conditional logistic regression

## Discussion

This study found that patients who utilized mental health services introduced by a case manager were less likely to be LTFU.

Previous studies have demonstrated that case management is associated with better retention and survival of patients with HIV [11,12]. However, case management has been differently operationalized and which specific intervention by case managers improve patients’ prognosis has not been fully elucidated [13,14]. What our result adds to this topic is that the mental health services based on a case manager’s assessment of each patient’s psychological aspects increased the chance for patients to receive regular care.

There could be several reasons for good retention of the patients who receive mental health services. Improved mental health as the result of the services could be related to good retention [15–17] and implementation of the services per se would be responsible for good engagement in care through making patients realize their own problems and tackling them. An understanding of a patient’s psychological aspects by the case manager and other medical staff members through communication with therapists would also contribute to better continuity of care. We cannot say that mental health service implementation directly reduced LTFU because of unmeasured confounding factors such as a patient’s attitude to accepting a case manager’s advice. However, our results suggested that patients who did not accept mental health services were more at-risk for LTFU.

Patients with HIV younger than 30 years of age or without regular employment were less likely to engage in regular hospital visits. These results agree with previous reports that showed the independent factors for LTFU were young age and low socioeconomic status [18–22]. Similar to previous reports [21,22], not taking antiretroviral therapy also strongly predicted LTFU. Patients with HIV who did not receive antiretroviral therapy were over seven times more likely to withdraw from regular care. They were speculated to be asymptomatic because, in our data, 93.2% did not have an AIDS diagnosis. Furthermore, because these patients were free from the side effects of antiretroviral drugs, they might not have felt an urgent need for strict regular checkups. We cannot determine the exact reason why some patients were not receiving antiretroviral therapy at the time of LTFU, because whether or not to start the therapy depends partially on individual preference. However, they were not assumed to be eligible or prepared for the therapy because most of them had CD4+ lymphocyte counts higher than the threshold for the therapy recommended by the guidelines of the time [23], or were within 90 days from their first visit.

This study has some limitations. First, because it is not a randomized-controlled study but an observational study, we cannot determine the causal relationship between mental health service induction and good retention. However, it is obviously ineffective to deliver patient-centered psychotherapy to patients without the need and it is unethical to withhold it from patients who need it. An observational study is reasonable to investigate the association between the implementation of psychotherapy and retention in actual clinical practice. Second, the survival of the patients was not investigated and we could not obtain the prognosis of LTFU cases. However, the growing number of studies including ones without complete follow-up systems, such as nationwide databases, indicates that LTFU is associated with worse survival, which can support the clinical validity of the present findings [24–27]. The third limitation is generalizability. Our findings would be applicable to HIV care facilities where case manager have good access to mental health services, but the availability, contents, and charge of mental health services differ among settings and influence results.

Our study has several strengths. All of our LTFU cases and controls received case management, unlike most previous studies that evaluated the effectiveness of case management. Consequently, we were able to demonstrate an association between retention and mental health service use as an element of case management more clearly. Therapeutic trends such as criteria for antiretroviral therapy initiation, pill burden, and drug side effects are always changing and they must have affected results; however, this bias was accounted for because we matched entry dates into the cohort and time spent in the cohort between LTFU cases and controls. We suspected that CD4+ lymphocyte count and antiretroviral therapy at cohort entry affect the subsequent continuity of care and we dealt with these factors as time-dependent variables. Thus, we could evaluate the influence of these factors at the time LTFU occurred more appropriately.

## Conclusions

Treatment of HIV has improved drastically, but care cannot be optimally offered if patients do not regularly keep hospital appointments. Paying attention to patients’ psychological problems and implementing adequate resources such as mental health services have significance in case management for better retention, and healthcare professionals must recognize that patients who do not utilize mental health services are at high risk of LTFU.
